# Hypoxia imaging and radiotherapy: bridging the resolution gap

**DOI:** 10.1259/bjr.20160939

**Published:** 2017-08

**Authors:** David Robert Grimes, Daniel R Warren, Samantha Warren

**Affiliations:** ^1^ Cancer Research UK/MRC Oxford Institute for Radiation Oncology, Gray Laboratory, University of Oxford, Old Road Campus Research Building, Off Roosevelt Drive, Oxford OX37DQ, UK; ^2^ Centre for Advanced and Interdisciplinary Radiation Research (CAIRR), School of Mathematics and Physics, Queen's University Belfast, UK; ^3^ Hall-Edwards Radiotherapy Research Group, Queen Elizabeth Hospital, Birmingham, UK

## Abstract

Oxygen distribution is a major determinant of treatment success in radiotherapy, with well-oxygenated tumour regions responding by up to a factor of three relative to anoxic volumes. Conversely, tumour hypoxia is associated with treatment resistance and negative prognosis. Tumour oxygenation is highly heterogeneous and difficult to measure directly. The recent advent of functional hypoxia imaging modalities such as fluorine-18 fluoromisonidazole positron emission tomography have shown promise in non-invasively determining regions of low oxygen tension. This raises the prospect of selectively increasing dose to hypoxic subvolumes, a concept known as dose painting. Yet while this is a promising approach, oxygen-mediated radioresistance is inherently a multiscale problem, and there are still a number of substantial challenges that must be overcome if hypoxia dose painting is to be successfully implemented. Current imaging modalities are limited by the physics of such systems to have resolutions in the millimetre regime, whereas oxygen distribution varies over a micron scale, and treatment delivery is typically modulated on a centimetre scale. In this review, we examine the mechanistic basis and implications of the radiobiological oxygen effect, the factors influencing microscopic heterogeneity in tumour oxygenation and the consequent challenges in the interpretation of clinical hypoxia imaging (in particular fluorine-18 fluoromisonidazole positron emission tomography). We also discuss dose-painting approaches and outline challenges that must be addressed to improve this treatment paradigm.

## INTRODUCTION

In 1953, Gray et al^1^ had observed that the concentration of oxygen in tissues markedly affects the response of animal tumours to radiotherapy. This finding has been well replicated up to the present day from a clinical perspective,^2^ with hypoxia associated with averse outcomes. In addition, oxygen can greatly modify the response of a patient to radiotherapy; for conventional X-ray therapy, regions of a tumour with high oxygen concentration are up to three-fold more amenable to treatment than anoxic regions. Hypoxia in tumours is something of a vicious cycle problem, with low oxygen level both promoting aggressive mutations and hampering treatment efficacy.

Radiotherapy itself has continued to evolve steadily since its introduction in the late 1800s.^3^ New treatment modalities such as intensity-modulated radiotherapy (IMRT) have emerged, which allow for the modulation of dose over small volumes, of the order 1 cm^3^. Parallel to this, functional imaging of tumour hypoxia is steadily becoming a clinical reality, raising the tantalizing prospect of delivering increased dose to hypoxic regions with heightened resistance. This concept is known as dose painting,^4^ where increased dose might be given to hypoxic subvolumes.

**Figure 1.**
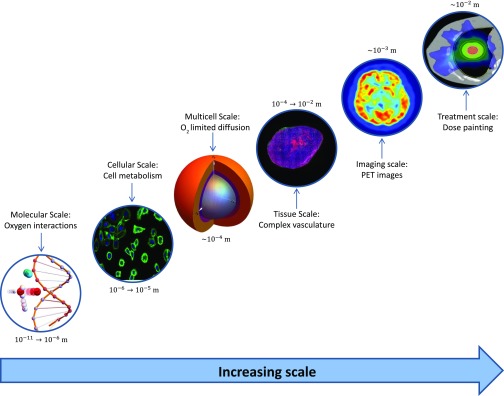
Oxygen-mediated treatment resistance as a multiscale problem.

Yet promising as dose painting is, there are still significant barriers to implementation of this promising modality. As depicted in Figure 1, hypoxic treatment resistance is inherently a multiscale problem—the best functional imaging of hypoxia is limited to the millimetre regime, whereas oxygen diffusion varies over a micron scale. Similarly, the impact of cellular oxygen consumption rate and vascular distribution can create exceptionally heterogeneous oxygen distributions. Even if hypoxia can be estimated, there is still much more work required in order to derive robust treatment plans for this eventuality.

If oxygen-mediated treatment resistance is to be overcome, these aspects cannot be considered in isolation. In this review, we consider the role of oxygen in radiobiological treatment resistance, the current state of *in vivo* functional hypoxia imaging and dose-painting methodology. In particular, we focus on the role of oxygen in treatment resistance and enhancement and functional imaging of *in vivo* hypoxia, as well as dose-painting work to date. Finally, we look at potential future work in this field and outstanding challenges that need to be surmounted.

## OXYGEN AND RADIOBIOLOGY

### Hypoxia and phenotype selection

Since Gray's initial observation in the 1950s, the deleterious consequences of hypoxia have been observed across numerous cancer types and sites.^5–7^ Under hypoxic conditions, tumour cells can respond to hypoxia by activating oxygen-sensitive signalling pathways, including hypoxia-inducible-factor pathways^8^ and the unfolded protein response.^9^ Although the precise mechanisms remain poorly understood, it is thought that these signalling pathways alter gene expression in an attempt to promote survival under adverse conditions, and ultimately allow cellular phenotypes to arise with evasive mutations, including the ability to metastasize. To compound this, hypoxia also drives angiogenesis, providing new routes for cancer cells to colonize,^10^ and affects cellular proliferation rates.^11^ The signals induced by microenvironmental hypoxia ultimately allow for a cascade of effects which eventually lead to the spread of tumour cells to distant sites.^12^ Thus, the oxygen microenvironment has a strong influence on how tumours will evolve and respond to treatment.

### Oxygen enhancement ratio

The advantageous effect of oxygen on treatment response is known as the oxygen enhancement ratio (OER), typically defined as the ratio of cell kill under well-oxygenated conditions relative to that under anoxia. Under anoxia, the OER is unity and the ratio increases with oxygen concentration. Although this can de defined in numerous ways, the most common formulation is given by

(1) $$\text{OER}=\frac{\text{Cell kill in oxic conditions}}{\text{Cell kill under anoxia}}$$

and typically has a maximum value of 2.7,^3^ suggesting that one would have to deliver 2.7 times the dose to an anoxic region to elicit the same level of cell kill. In reality, OER is not linear with oxygen concentration, instead OER yields a curve where half-maximum level manifests around 2.5–3 mmHg. The effect quickly saturates, obeying a roughly hyperbolic relationship with oxygen partial pressure.^3^ Beyond partial pressures of 20 mmHg, no additional treatment benefit is garnered by increasing oxygen concentration. Furthermore, oxygen must be present at irradiation or microseconds thereafter, as adding oxygen subsequent to this does not improve therapy response.^3,13^

**Figure 2.**
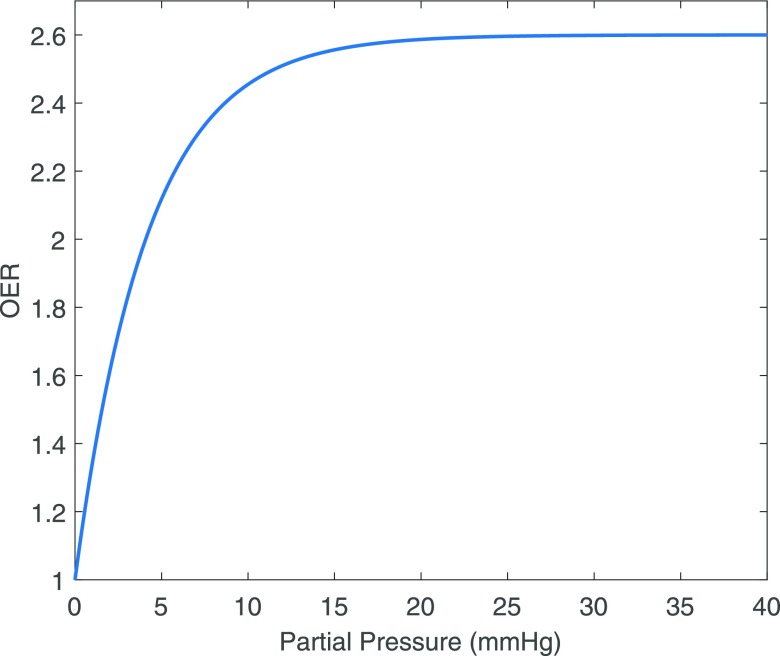
A typical oxygen enhancement ratio (OER) curve, saturating at *p* > 20 mmHg.

This OER curve is depicted in Figure 2 and has generally been described empirically by approximation with hyperbolic functions^14^ since the late 1950s. Although an equation of this form describes the behaviour of the oxygen curve well, it does not posit any mechanism of action for what has been observed. One likely radiochemical rationale for this phenomenon is oxygen fixation hypothesis, which postulates that while most deoxyribonucleic acid (DNA) can be repaired following radical damage, the presence of molecular oxygen with radical species produces a reactive agent whose damage is more difficult or impossible to reverse.

**Figure 3.**
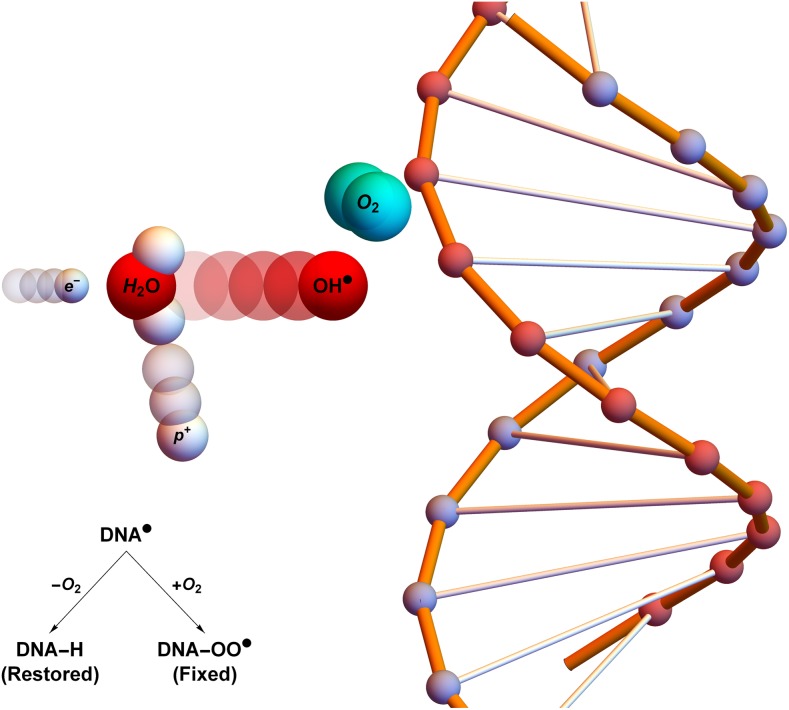
Oxygen fixation hypothesis: a high-energy electron created by an X-ray photon (*e*^−^) impinges on a water molecule, liberating a proton (*p*^+^) and creating a hydroxyl radical (OH˙). This reactive molecule then impacts on deoxyribonucleic acid (DNA˙), resulting in ionization damage, DNA. This can be readily repaired to its original state (DNA-H), but in the presence of molecular oxygen, a peroxy radical is formed (DNA-OO˙), fixing damage into a permanent irreparable state. Taken from Grimes and Partridge with permission from Institute of Physics (IOP).^16^

Under this schema, DNA damage from incoming high-energy photons can be caused in a number of ways—direct damage arises when the photon interacts with DNA, ionizing the molecule *via* Compton scattering. But more commonly, incoming photons may interact with other matter, typically water. In these ionization events, a high-energy electron is liberated. These charged particles may impinge on other water molecules, creating highly reactive hydroxyl radicals (R˙). This damage can be readily chemically repaired, but if such radicals encounter an oxygen molecule, they may combine to form a peroxyl radical $\left({\text{RO}}_{2}^{\cdot }\right)$. This damage is much more difficult for the cell to repair^15^ and is illustrated in Figure 3. Thus, the presence of molecular oxygen can “fix” damage in an irreparable state.^3,15^

The mechanisms underpinning OER have been the subject of more recent work,^16^ taking the first principles rather than empirical approach. This mechanistic treatment uses Poisson statistics to estimate the likelihood of an interaction between ionized DNA and an oxygen molecule, taking into account a number of vital physical parameters, including the thermal velocity and mean free path of oxygen molecules, the interaction volume and the availability of oxygen per unit volume. Under this schema, OER is a function of oxygen partial pressure *p* given by

(2) $$\text{OER}\left(p\right)=1+\frac{\phi_{\text{O}}}{\phi_{\text{D}}}\left(1-e^{-\varphi p}\right)$$

where *p* is the oxygen partial pressure in mmHg, $\phi_{\text{O}}/\phi_{\text{D}}$ is the ratio of cells killed by oxygen fixation to those killed directly and *φ* is a parameter derived from the first principles. This model yields the familiar OER curve and fits well to a range of experimental data, including human cells, bacteria and yeast. Despite the significant biological differences between these subjects, this analysis suggests consistent values of $\phi_{\text{O}}/\phi_{\text{D}}=1.6\pm 0.03$ and *φ* = 0.26 ± 0.02 mmHg^−1^ for conventional photon treatments. This apparent biological invariance strongly supports the contention that OER arises from radiochemistry, and this analysis provided further evidence that the oxygen fixation hypothesis is indeed the mechanism responsible for the observed boosting effect of oxygen on radiotherapy.

#### Variation of oxygen enhancement ratio with linear energy transfer

It is important to note that the maximum OER obtainable varies with the energy of the radiation used. Experimental evidence suggests that high-energy charged-particle radiation has a markedly lower maximum obtainable OER, and some empirical functions exist to describe this reduction in OER with increasing linear energy transfer (LET).^17^ The reason for this is likely the relatively intuitive conclusion that with increasing LET, direct damage becomes a more dominant process, and there is correspondingly less excess damage due to oxygen fixation. This suggests that for increasing LET, ϕOϕD approaches zero, with direct damage dominating at high enough energy.

### Factors influencing oxygen availability

#### Diffusion limited hypoxia

As oxygen diffuses from a source, it is consumed by cells around it and, eventually, the oxygen concentration diminishes to zero. In healthy tissue, this is well regulated and cells tend to have adequate oxygen. In cancer, cell growth is abnormal and, as a consequence, the oxygen supply is frequently inadequate, leading to chronic hypoxia beyond the diffusion distance *r*_n_. Experimentally, diffusion distance in tumour tissue has been measured at between 100 and 200 μm.^18–23^

The oxygen consumption rate (OCR) is of paramount importance in this process, and cells with a higher OCR have much shorter diffusion lengths. The precise relationship between OCR and diffusion limit depends on several factors, chief among them being source geometry. Early mathematical frameworks modelled vessels as cylindrical emitters,^18,19,24^ a formulation known as the Krogh model. In limited circumstances, this can work well, but many of its assumptions (such as strictly radial diffusion) do not hold in complex tumour tissue.^25^

**Figure 4.**
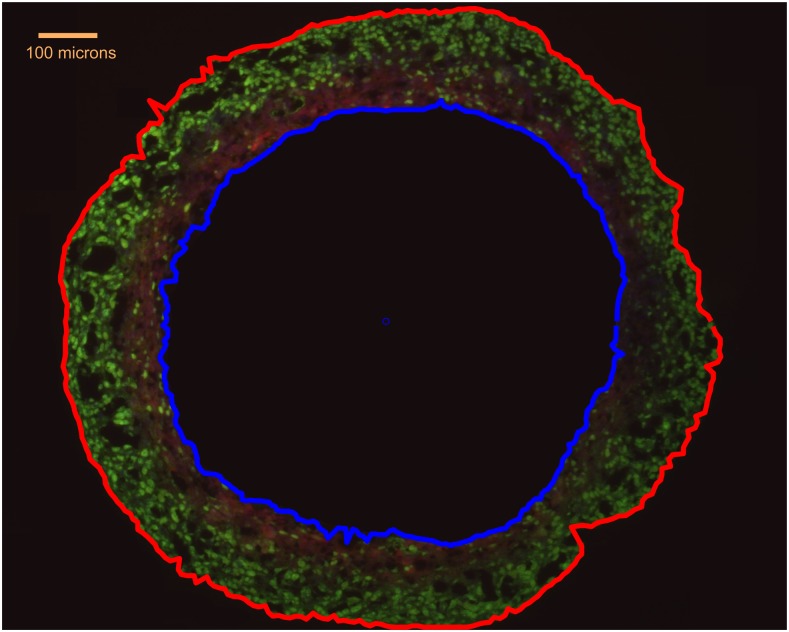
A DLD1 tumour spheroid, with external boundary marked in red. The oxygen-limited anoxic core (blue outline) is also shown. Green staining is the ki-67 proliferation marker and red is the hypoxia marker EF5. Adapted from Grimes et al with permission from Royal Society Interface.^23^

Part of the complication is that vascular environments in tumours tend to be highly heterogeneous. One alluring experimental model for investigating the relationship between OCR and diffusion limited hypoxia is to instead use tumour spheroids. These are three-dimensional aggregates of cancer cells, with metabolic profiles more similar to *in vivo* tumours than simple monolayers.^26^ If sufficiently small, spheroids grow exponentially initially, forming anoxic cores when they grow beyond the diffusion distance of oxygen.^27^ A typical spheroid is illustrated in Figure 4. The spherical geometry lends itself to analytical solutions,^23^ and it can be shown that the diffusion limit of a spheroid *r*_l_ is related to its OCR, *a*, by

(3) $$r_{\text{l}}=\sqrt{\frac{6Dp_{\text{O}}}{a\Omega }}$$

where *p*_O_ is the oxygen tension at the spheroid surface, *D* the diffusion constant of the tissue and *Ω* = 3.0318 × 10^7^ kg mmHg m^−3^, a constant arising from Henry's law. When spheroids grow beyond a radius of *r*_l_, they begin to develop anoxic cores. Analytical relationships between the extent of the viable rim and anoxic core, as well as the oxygen distribution have been previously derived and validated. These extents are closely linked to cellular OCR, which strongly influences the maximum dimensions that a spheroid can obtain.

It is worth noting that tumours may have highly complex geometries and, in general, one cannot readily apply neat geometries to the *in vivo* case, but these models give us some valuable insight into how OCR shapes oxygen distribution throughout the tumour environment. In these formulations, OCR is generally considered to be a constant, but it is also possible to formulate oxygen consumption as a function of oxygen availability, typically obeying Michaelis–Menten-like kinetics. Even if this is assumed, the resulting oxygen distributions show only negligible difference from the constant consumption case.^29^

#### Vasculature and perfusion

Tumours typically display highly contorted and chaotic vasculature, and these tortuous and irregular vessels lack the hierarchical arrangement of healthy vessels.^30–36^ In addition to this, endothelial cells in tumours tend to be abnormal^32^ and the secretion of angiogenetic growth factors^10^ encourages the formulation of bizarre vessel structures. In some instances, these microvessels are only perfused by plasma or simply not perfused at all,^2,36^ therefore despite the presence of a vessel, there may be no oxygen supplied to the tumour region. In this environment, hypoxia can readily become dominant even when there is apparent vascular supply. In addition, perfusion may vary temporally, leading to regions of acute hypoxia.^37^ Although the evidence is currently unclear, there is also some suggestion that chronic hypoxia (limited by diffusion) and transient hypoxia might have intrinsically different radiosensitivity profiles due perhaps to changes in the repair capacity of cells chronically starved of essential nutrients, particularly oxygen.^38,39^ This remains an open question, with further studies needed to explore it more deeply.

## SPATIAL MEASUREMENTS OF OXYGENATION

A number of methods have been developed to measure or infer tissue oxygenation *in vivo*. Direct, invasive physical measurements of oxygen concentration may be performed, *e.g.* using polarographic electrodes^40^ or fluorescence probes.^41^ However, electrode responses are non-linear functions of the oxygen distribution within the sensitive volume (typically 100 μm in diameter), affecting their ability to detect extreme values of the oxygen distribution.^42,43^ There are also considerable challenges in sampling a sufficiently large proportion of the tumour in a spatially precise manner, as would be required to inform a prescription for radiotherapy dose painting (although direct spatially localized measurement of oxygenation has been demonstrated in animals^44^).

Imaging constitutes an alternative framework in which measures of oxygenation can be localized in three dimensions. Image contrast relating directly to deoxyhaemoglobin concentration in blood can be obtained using gradient-echo MRI;^45^ changes in concentration can be induced by manipulating the inhaled gas mixture (the blood-oxygen-level-dependent effect), enabling qualitative assessment of intravascular PO_2_.^46–48^ Although BOLD-MRI methods provide information on oxygen sources within the tumour, this is not sufficient to reliably infer the oxygen concentration far from vessels (which also depends heavily on oxygen consumption in particular). There has instead been considerable focus on optimizing molecular hypoxia imaging, which measures the concentration of a tracer molecule with oxygen-dependent binding characteristic. In this paradigm, the signal has a biological (as opposed to physical) interpretation and may arise anywhere in tissue, assuming adequate tracer delivery.

### Molecular hypoxia imaging

For biological hypoxia imaging to be effective, it is necessary to detect very low concentrations of a molecule in tissue: positron emission tomography (PET) and single-photon emission CT are therefore used to image the distribution of a radiolabelled tracer, having good specificity for detection due to low background radioactivity in the human body. A wide range of tracers have been proposed for clinical use in both modalities and have been reviewed in detail elsewhere.^49,50^ The following discussion is given in the context of a PET-based approach, which currently offers better sensitivity,^51^ and often better spatial resolution in the clinic than single-photon emission CT; however, many of the points raised will be relevant to all molecular imaging.

In PET-based hypoxia imaging, image contrast arises from the decay of a radiopharamaceutical (tracer), delivered to tissue *via* the bloodstream. Existing research has predominantly focused on two classes of hypoxia tracers. The first of these is Cu-ATSM, labelled with any of copper's four long-lived positron-emitting isotopes (60, 61, 62, 64); whilst the hypoxia-specific binding characteristic and desirable uptake and washout properties have been demonstrated both *in vitro* and *in vivo*, the detailed mechanism for retention has not yet been conclusively determined.^52^ The second class of tracers consists of fluorine-18-labelled (^18^F) nitroimidazoles and includes ^18^F-fluoroazomycin-arabinoside (^18^F-FAZA), 2-(2-Nitro-1H-imidazol-1-yl)-N-(2,2,3,3,3-[^18^F]pentafluoropropyl)-acetamide and ^18^F-fluoromisonidazole (^18^F-FMISO); these molecules bind to intracellular macromolecules as a result of two reduction processes, the first of which may be reversed in the presence of oxygen.^53^ We will discuss nitroimidazoles, in particular the tracer ^18^F-FMISO, in more detail.

#### Static hypoxia positron emission tomography imaging

Comparison of the length scales for oxygen gradients in tissue (100 μm) and the typical resolution of clinical PET (4 mm) highlights the potential for considerable heterogeneity of oxygenation within an image voxel. The image signal observed will be an average measure of uptake in the entire voxel, but two features of this process complicate the interpretation of hypoxia PET: the non-linearity of the signal as a function of local PO_2_ and the variation of the signal as a function of cellularity.

##### Non-linearity

**Figure 5.**
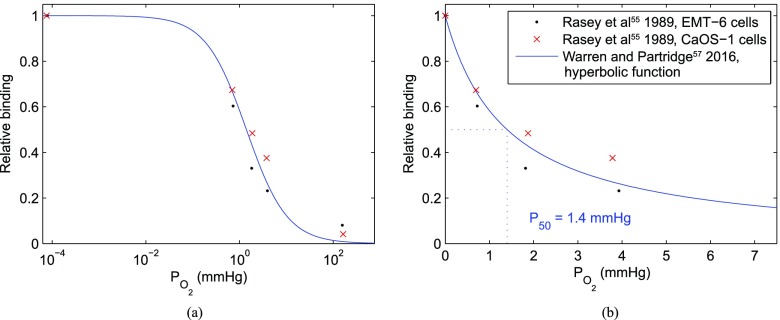
Illustration of the non-linear binding relationship for fluoromisonidazole (FMISO) as function of PO_2_. The points show experimental data from Rasey et al,^54^ and the line shows a hyperbolic functional form fitted to autoradiographic data from tumour spheroids.^56^ (a) Logarithmic axis; (b) linear axis at low PO_2_.

In cellular studies, ^18^F-FMISO binding has been found to display a sharp (but not instantaneous) increase as local PO_2_ decreases. Half-maximal uptake values are reported in the range 0.8–2.1 mmHg,^54–56^ and the functional form of the relationship is illustrated in Figure 5. It might therefore be suggested that in the absence of other confounds, ^18^F-FMISO is indicative of the cellular “hypoxic fraction”, where hypoxia is defined as ≤2 mmHg. However, since the binding function is smooth, rather than a simple threshold, and is also non-linear, a number of scenarios can lead to the same mean uptake value in a voxel. In Figure 5, it can be seen that approximately the same signal would be expected if 25% of a voxel was anoxic (but viable) and the remainder well oxygenated, or if the voxel is 50%/50% split between 1.4 mmHg and oxic, or if the whole voxel is at 4.2 mmHg. The radiobiological response would be likely to differ markedly between these scenarios, as illustrated in Figure 2.

##### Cellularity

Since the proposed mechanism for nitroimidazole binding requires an active electron transport chain, no specific binding would be expected in areas of necrosis or acellular material.^53,57,58^ A reduction in misonidazole (MISO) uptake has indeed been demonstrated in autoradiographic studies of spheroids with necrotic cores,^59–61^ which develop due to anoxia, and similar effects have been seen in necrotic regions of tumours in rats using ^18^F-FMISO and ^18^F-fluoroerythronitroimidazole autoradiography.^62^ In the case of glioblastoma, high ^18^F-FMISO uptake has been shown as a predictor of micronecrosis in humans.^63^

At the clinical scale, subvoxel necrosis may reduce the maximum signal achievable and cause it to occur at higher PO_2_. Simulation studies illustrate the possible non-monotonic relationship between average voxel oxygenation and ^18^F-FMISO uptake^56,64^ in the presence of subvoxel necrosis. Significant proportions of other biologically inert substances within a voxel such as air, fluid, mineral bone and connective material might be expected to confound the hypoxia signal in a similar manner.

**Figure 6.**
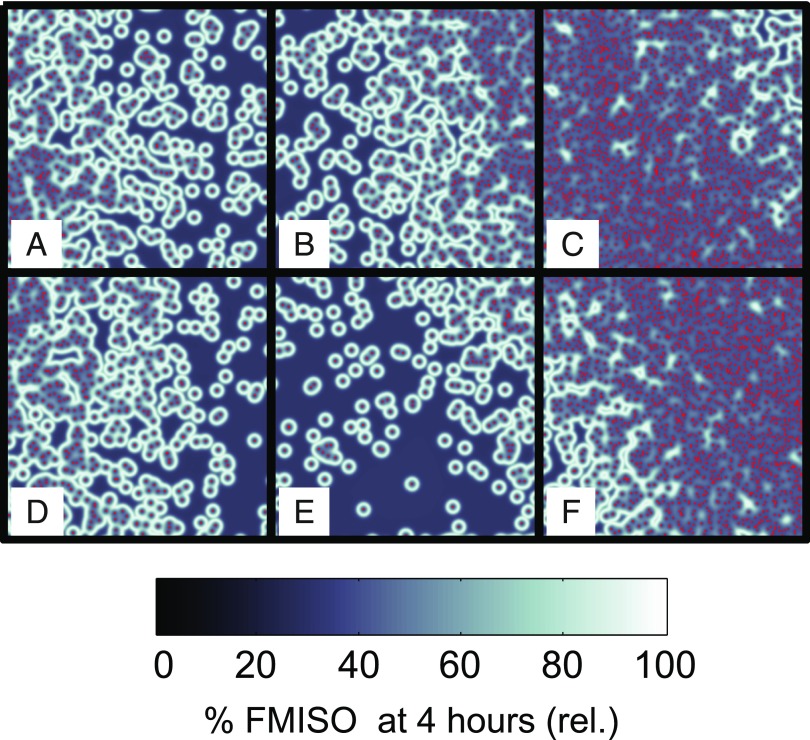
A simulated mean oxygen and fluoromisonidazole (FMISO) uptake in 4-mm voxels (a–f) using a two-dimensional vessel map with varying vascularity, calculated by the methods described in Warren and Partridge.^56^ Red points indicate simulated blood vessel positions. Voxels (e) and (f) are predicted to show very similar binding, despite a large difference in PO_2_, due to the extent of necrosis in voxel (e).

Both of these effects have been illustrated well in pre-clinical comparisons of ^18^F-FAZA autoradiography and histology^65,66^ and also in computational simulations of FMISO in relation to PO_2_ (Figure 6). Further biological complications may also exist; for example, there are suggestions that the critical PO_2_ for nitroimidazole binding varies somewhat with cell/tumour type.^67,68^ Overall, static PET images are unlikely to provide sufficient information to quantify hypoxia in all scenarios.^69^ Additional information, *e.g.* in the form of tracer uptake dynamics, may resolve this situation.

#### Dynamic hypoxia positron emission tomography imaging

The general effects of necrosis and the non-linear ^18^F-FMISO binding relation are to introduce difficulties in distinguishing between voxels with good oxygenation and those with very poor oxygenation. Perfusion would be expected to vary considerably between these two scenarios and, since the tracer is administered in the bloodstream, could be assessed by examining images acquired at multiple time points after tracer administration (dynamic analysis). Furthermore, the slope of the time activity curve may contain additional information regarding the fraction of cells which bind tracer.

Simulations have shown that the ratio of the “late” ^18^F-FMISO activity (mostly bound tracer, acquired 4 h post injection) to “early” activity (mostly perfusion, acquired during the first 15 min) result in a much better correlation between image signal and average tissue oxygenation.^64^ It is possible to identify characteristic time activity curves for vasculature, hypoxia and necrosis,^70^ but (as with a static analysis) all these features may be present in varying proportions within a voxel. A wide range of kinetic modelling has been carried out to estimate the relative contribution of some or all of these features in a given region.^71^ Thorwarth et al^72^ developed a detailed kinetic model that allows prediction of the signal that originate in hypoxic cells for each voxel; pharmacokinetic modelling has also been used to isolate maps of the hypoxia-specific binding constant.^73^ A study comparing dynamic PET analysis to image-guided PO_2_ measurements in animals has suggested that it enables better discrimination of hypoxia than static PET analysis.^74^

## DOSE PAINTING

The customization of radiotherapy prescriptions based on spatial information from hypoxia imaging is known as dose painting. In principle, this allows selective boosting dose to radio-resistant regions. The ability to dose paint has been demonstrated using a number of advanced radiotherapy techniques, including fixed field IMRT, volumetric modulated arc therapy, tomotherapy and proton therapy.^75–78^ These studies indicate that complex non-uniform dose distributions can be optimized and delivered with a range of techniques, with broadly equivalent target coverage and dose conformity. However, proton therapy reduces integral dose to the entire patient.

Two separate paradigms have been proposed for translating hypoxia images into dose prescriptions: dose painting by contours (DPBC) and dose painting by numbers (DPBN).

### Dose painting by contours

In DPBC, a threshold value for the boost volume is selected based on, *e.g.* tumour-to-blood uptake ratio (T : B) ≥1.3 for FMISO,^79^ standardized uptake value (SUV) ≥1.4 for Cu-ATSM^80^ or SUV >50% mean muscle SUV for FAZA.^81^ Most studies so far have taken this approach, as it is generally easier to implement into conventional clinical workflow using commercial treatment planning software. DPBC also (in general) produces less steep dose gradients or more contiguous/uniform dose boost regions, which might make these dose distributions slightly easier to create and deliver and more robust to spatial errors.

Several authors have looked at the value of using multiple tracers [^18^F-fludeoxyglucose (^18^F-FDG), hypoxia or proliferation] to identify boost regions within the target volume,^80,82–86^ although the conclusion seems to be that there is little correlation between the boost volumes delineated by each individual tracer, as they are designed to identify different characteristics within the tumour volume. Clausen et al^87^ compared delineating either the “union” or the “intersection” of ^18^F-FDG and FMISO volumes, finding that the “union” volumes are larger and, in some patients, perhaps too large to be safely used for dose escalation.

Most of the FMISO dose-painting work in the current literature concerns head and neck cancers, in particular planning studies involving a small number of patients. Typical threshold values for FMISO are T : B 1.3,^79,88,89^ and dose escalation is achievable for the vast majority of cases. For example, Choi et al^89^ showed the feasibility of dose escalation from 72 Gy (2.4 Gy per fraction) to 78 Gy (2.6 Gy per fraction) in 6 of 8 patients; Hendrickson et al^90^ achieved a planned 10-Gy boost for every patient in a cohort of 10, predicting a tumour control probability (TCP) increase of 17%. Less satisfactory planning results were seen when the segmented high-dose boost volume is too small to achieve adequate dose coverage^80^ or when the tracer signal occurs as multiple diffuse boost volumes.^81^ A clinical trial is currently open-investigating FMISO dose painting in head and neck cancer (NCT02352792).

Other tumour sites of interest studied using FMISO include the rectum^84^ and pancreas.^86^ The rectum shows non-specific FMISO uptake and diffusion through the bowel wall. The reported seven patients with pancreatic cancer showed no correlation between typical PET quantification metrics and tumour size, and hypoxia was only visible in two patients' FMISO PET scans. Early reports have also demonstrated the feasibility of hypoxic dose painting using FMISO in the lung.^91,92^

### Dose painting by numbers

DPBN is an alternative approach to dose painting, whereby a prescription dose is individually calculated for every voxel of a tumour, based on local biological information from multimodality imaging. Alber and Thorwarth^82^ described an elegant (theoretical) framework, which could produce DPBN prescriptions that are robust to imaging uncertainties. Sterpin et al^93^ showed that such uncertainties may be addressed by blurring and dilating the prescribed dose, accounting for random and systematic errors, respectively, without the need for explicit consideration by the treatment planning system. The theoretical work by Thorwarth et al^94^ compared a uniform dose boost and DPBN and found that DPBN results in a much higher predicted improvement in TCP than a simple uniform boost. DPBN schemes have been proposed whereby dose is escalated as a linear function of image intensity, capped at various minimum and maximum image intensities.^94–97^

Several groups have suggested “dose redistribution” rather than dose escalation, since any improvement in TCP calculated for the non-uniform dose plan will be “true” rather than just a result of any increase in mean dose.^75,76,80,87,98–100^ Most of these authors used commercial treatment planning systems. Malinen et al^98^ segmented the tumour in a canine subject into four compartments according to the level of hypoxia, each with a calculated TCP. The dose distribution was then designed to maintain constant mean dose to the entire target volume but with each compartmental dose optimized to improve TCP. Systematic errors in dose were less critical than random errors (where voxels were randomly assigned to the “wrong” hypoxic compartment). Søvik et al^99^ further showed that non-uniform dose was better than uniform dose but that adaptive re-planning could further improve TCP over the course of an 18-fraction treatment. Daily adaptive re-planning was no better than re-planning twice weekly. Toma-Dasu et al^101^ also examined a voxel-based dose prescription method with dynamic and static hypoxia to achieve a TCP of 95% (for which the dose prescription varied: 65–121 Gy). Bowen et al^102^ examined different methods of translating the hypoxia histogram into a dose prescription based on either a polynomial or sigmoidal function.

A variety of hypoxia imaging techniques have been explored as a basis for dose painting, including dynamic contrast enhanced MRI (DCE-MR) and PET with the tracers Cu-ATSM, ^18^F-FAZA and FMISO. Very useful information on treatment planning and plan evaluation is also obtained from work using ^18^F-FDG or ^18^F-FLT (^18^F-3-deoxy-3′-fluorothymidine) for boost volume delineation. The phase I safety trial for DPBN adaptive planning for head and neck cancer describes a neat way of planning radiotherapy treatments with complex prescriptions and multiple time point planning scans.^76^ Each patient receives 32 fractions delivered using 3 different treatment plans. Plan 1 (fractions 1–10) is DPBN using the first functional imaging scan (in the trial, this had two different dose levels), Plan 2 (fractions 11–21) is DPBN using the second functional imaging scan and Plan 3 (fraction 22–32) is a uniform IMRT plan. The largest planning studies^76,96^ (involving 21 and 20 patients, respectively) have used ^18^F-FDG as a tracer.

## DISCUSSION AND CHALLENGES

Oxygen has a substantial impact on treatment response, and hypoxia presents a serious impediment to therapy by increasing radioresistance. In addition, a low oxygen microenvironment is highly correlated with the development of metastatic phenotypes and poor prognosis. Hypoxia is a selection pressure for the evolution of phenotypes with the capacity to both endure harsh environments and crucially to migrate beyond the bounds of the tissue from whence they arose.^10^ These clones have the capacity to proliferate and survive in hypoxic environments,^12^ and while the exact interplay is still an area of active study, the negative influence of hypoxia on prognosis has long been appreciated.^1^ As the microscopic oxygen distribution is highly heterogeneous, it is notoriously difficult to characterize *in situ*. Functional imaging using hypoxia tracers such as FMISO PET may be suitable for determining regions of substantial hypoxia. This information could then be used in conjunction with OER data to prescribe a higher dose to low oxygen regions and thereby help alleviate or overcome hypoxia-mediated treatment resistance.

The experimental evidence to date suggests that in test–retest repeatability, 70–80% of the hypoxic volume is stationary, although a small transient component is also seen in some patients.^103–107^ This makes boosting target volume definition using baseline hypoxia imaging feasible in principle. Modelling results also suggest that short-time-scale oxygenation variations do not affect the formation of FMISO image contrast,^56^ although much more investigation is required to answer these questions rigorously. If multiple images are obtained, it might be possible to gauge the patient-specific hypoxia stability, and if this is taken during treatment, it may lead to the possibility of biologically adaptive treatment protocols, although much more experimental data would be required to ascertain whether this avenue has potential.

**Figure 7.**
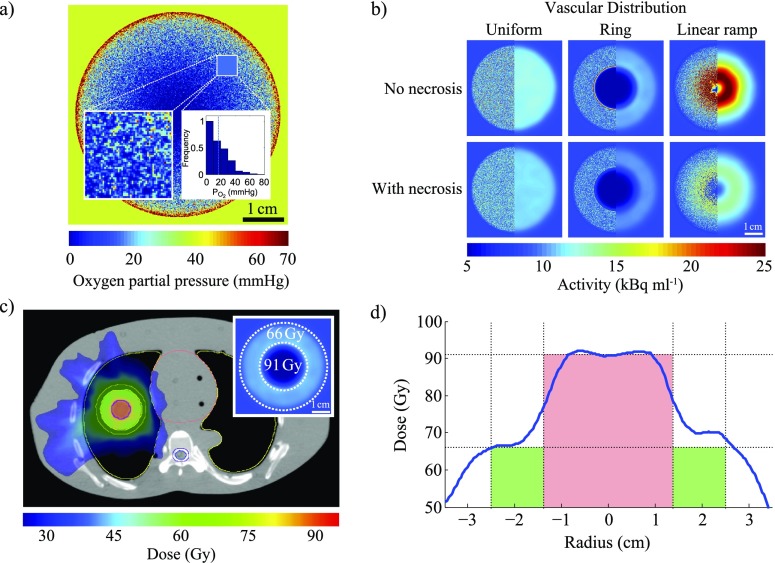
(a) A simulated microscopic oxygen distribution in a 5-cm diameter spherical tumour; *inset*: heterogeneity within the region with size equivalent to a positron emission tomography voxel (map and histogram, dashed line represents mean). (b) A simulated fluoromisonidazole (FMISO) distributions for a spherical tumour under different assumptions (see text: Discussion and Conclusions, paragraph 3 for details). (c) Planned volumetric modulated arc therapy treatment. (d) An example line profile of planned dose through the gross tumour volume, plotted against prescription (green: vascularized rim; red: boost to hypoxic core).

Hypoxic dose painting shows much promise and has been experimentally demonstrated in a number of studies, several of which are discussed in this review. An example of the stages and scales that must be considered to develop a dose-painting strategy is illustrated in Figure 7, which describes an *in silico* model of a lung tumour. This comprises a sphere positioned inside the lung of an anthropomorphic phantom, with locally varying oxygen distributions calculated at 100 μm resolution, using previously published methods^36^ and a varying vascular density function. Radial vascular density profiles are either uniform, a step function (creating a ring around a centrally avascular tumour), or ramped (increasing linearly with radius), as shown in Figure 7b. Local FMISO uptake at 4 hours post injection was predicted using a model of tracer-binding kinetics,^56^ which includes hypoxic necrosis. This was subsequently convolved with a 4.5-mm full-width-at-half-maximum Gaussian to simulate a measured PET image, which is shown in the right half of each panel of Figure 7b. Representative prescription doses were calculated for an image-derived hypoxic boost region, and dose distributions were created using a commercial treatment planning system (Eclipse^™^ v. 13), assuming realistic normal tissue constraints. Although this process is possible in a computer simulation where microscopic oxygen distribution is already “known”, the necessary information is not readily obtainable in the clinic. Substantial biological and mathematical research must therefore be carried out before a similar mechanistic approach could be implemented in practice.

Although the ability to deliver selective dose boosts to discrete spatial regions is within our means, a significant number of outstanding questions and unresolved challenges remain. Some major issues are enumerated below:

1. *Imaging resolution*: Functional hypoxia imaging holds much allure, but it is important to note that the physics of PET imaging systems limits their resolution to the millimetre domain. As oxygen diffusion occurs over a micron scale, and tumour oxygen distributions are highly heterogeneous *in vivo*, the distribution of oxygenation within a measured voxel may not be adequately represented by a single image value and simple interpretation may be potentially misleading.
2. *Biological interpretation of images*: The underlying assumption of dose painting is that it is possible to inform radiation prescriptions based on the information derived from imaging, but there are several potential complications. As illustrated in Figure 6a–f, factors such as necrosis might confound the analysis of functional imaging data. There is some evidence to suggest that different cell types have varying FMISO binding characteristics, which itself would potentially skew understanding.^56^ Certainly, OCR has been demonstrated to differ significantly between cell types,^28^ impacting oxygen distribution. As *in situ* tumours are highly biologically heterogeneous, there may be different binding characteristics and OCRs even within the same tumour.
3. *Mathematical modelling*: Because of the physical limitations of functional imaging and the inherent biological variation even within tumours, more mathematical modelling is urgently required to bridge the scale gap between that which we can image and the most likely underlying biology. The scale gap between what can be imaged and the oxygen diffusion scale spans up to three orders of magnitude, and there is no current method for accurately determining the most likely underlying oxygen distribution in a voxel. To address this, robust biologically informed mathematical models are essential
4. *Dose delivery*: Even if it were possible to produce an ideal prescription dose distribution accounting for all the above complications, constraints are imposed by existing dose delivery techniques. Such limitations include the maximum deliverable dose gradient and the accuracy of patient positioning. Furthermore, without daily treatment adaptation, changes in patient anatomy, such as tumour shrinkage and weight loss, will also introduce uncertainties that must be accounted for in the final treatment plan.

In relation to imaging resolution, it is highly unlikely that this can be significantly improved for PET imaging due to fundamental physical constraints. Unless new modalities circumvent this difficultly, hypoxia image information will remain intrinsically limited and biological interpretation will be difficult. For example, the inset of Figure 7a depicts a histogram taken from a known underlying microscopic oxygen distribution, with a mean of *p* = 20 mmHg. However, this mean is potentially misleading because a significant portion of the volume lies below this and therefore the single voxel-derived value may be skewed. The question of how to translate from a functional hypoxia image to a dose prescription remains largely unanswered. Further biological research is needed to help inform this, and in particular, new mathematical models are urgently required to bridge the resolution gap. These models are vital if we are to estimate the most likely underlying distributions and deliver dose accordingly, but they remain challenging due to the complex biology of the tumour microenvironment and the inverse nature of the problem at hand.

Dose delivery issues may be overcome to some degree by recent technological developments. Spot-scanned proton and heavy ion therapies have the potential to deliver steeper dose gradients than intensity-modulated photon approaches but introduce additional considerations in the form of range uncertainties. Adaptive treatments are already in routine clinical practice in many centres but are rarely backed up by repeat biological imaging. ^18^F-FMISO PET, in particular, requires considerable clinical resource and is burdensome for the patient, therefore is unlikely to be suitable in this situation. Alternative approaches may be necessary for daily biological adaptation, and the MR-linear accelerator offers promise but will require its own detailed characterization from physical and biological perspectives before optimal protocols can be developed. In the studies cited in this work, dose escalations of up to 15% were reported, but it is likely that much higher increments would be required for clinical significance. Modern radiotherapy interventions may make 100- to 120-Gy doses achievable in some circumstances, whereas equivalent doses can definitely be achieved using stereotactic treatment. However, there may be no need to go quite this high; mechanistic modelling of re-oxygenation suggests that more moderate boost doses may be sufficient to obtain early re-oxygenation and cure.^108,109^ The requisite level of dose boosting to radioresistant hypoxic regions remains an open question.

This review has chiefly focused on presenting an overview of tumour hypoxia and its detrimental consequences. Although the concept of boosting dose to radioresistant regions has been elucidated in some depth, it is important to note that this is certainly not the only viable strategy to overcome hypoxia. Chemical interventions might play a sizable role in tackling hypoxia-induced treatment resistance. These include strategies including breathing hyperoxic gas with compounds such as carbogen, and hypoxic-specific cytotoxic agents. There is also a growing role for hypoxic sensitizers such as nimorazole. Some of these agents derive their efficacy from moderating oxygen consumption rate and thus reducing hypoxia—gemcitabine, *e.g.* is a well-known radiosensitizer and recent investigations have suggested that it markedly decreases oxygen consumption in treated cells^28^ thereby countering hypoxia. Although beyond the scope of this review, it is important to note the promising potential of chemical agents to address clinical hypoxia.

## CONCLUSION

Tumour oxygenation is of paramount importance to both treatment efficacy and patient prognosis. Overcoming the negative effects of hypoxia is a complex and deeply interdisciplinary problem and will take concerted research effort from interface fields to resolve. In particular, the multiple spatial scales make it a challenging problem, and these resolution gaps must be bridged to maximize the effectiveness of dose painting. Nevertheless, pragmatic approaches have already been demonstrated to be feasible and serve as a promising foundation on which future research can be built.

## FUNDING

This work had been supported by Cancer Research UK under grant numbers C5255/A15935 and C53469/A19834.
